# *The safe practitioner* framework: an imperative to incorporate a psychosocial sub-curriculum into dental education

**DOI:** 10.1038/s41415-024-8231-9

**Published:** 2025-03-28

**Authors:** Luke J. Dawson, Kathryn Fox, Marina Harris, Mark Jellicoe, Callum C. Youngson

**Affiliations:** 41415334239001 Professor of Dental Education, School of Dentistry, Faculty of Health and Life Sciences, University of Liverpool, Liverpool, UK; 41415334239002 Honorary Senior Lecturer, School of Dentistry, Faculty of Health and Life Sciences, University of Liverpool, Liverpool, UK; 41415334239003 Associate Professor of Dental Education and Wellbeing, School of Dental, Health and Care Professions, Faculty of Science, University of Portsmouth, Portsmouth, UK; 41415334239004 Senior Lecturer, The University of Law, Science School (Psychology), Leeds, UK; 41415334239005 Emeritus Professor, School of Dentistry, Faculty of Health and Life Sciences, University of Liverpool, Liverpool, UK

## Abstract

A primary aim of dental schools is to produce competent and caring independent professionals, capable of developing themselves and serving the needs of their patients through reflective practice and self-regulated continuous learning. The General Dental Council has also explicitly recognised the importance of self-regulated learning, and other associated behaviours, in the new *The safe practitioner* framework. However, traditional learning designs focus on the development of academic and clinical skills, and assume that psychosocial skills, which support self-regulated learning and enable the management of personal challenging circumstances, are already present. Unfortunately, data suggest that the psychosocial skills in many students currently entering healthcare programmes are relatively underdeveloped, impacting upon their approaches to learning and their mental health, and potentially, patient safety. Therefore, there is a need to support students in their psychosocial development. This development starts with teachers understanding the societal, academic and environmental circumstances that their current students have experienced, followed by the consideration of the importance of psychosocial skills within their dental education. This paper discusses these matters and suggests a psychosocial sub-curriculum along with a suggested framework for its implementation.

## Key points

- Discusses the need for an introduction of a psychosocial curriculum within dental education to address the needs of the current generation of dental students and meet the General Dental Council's required outcomes.
- Proposes a syllabus and organisation of subjects to allow dental education providers to facilitate monitoring of satisfactory student development in this area.
- Considers the change to *The* *safe practitioner* framework as an opportunity to develop key behaviours that can support learning, personal well being, and good long-term mental health.

## Introduction

In 2024, the General Dental Council (GDC) released its *The safe practitioner* framework (SPF)^1^ which will replace the existing *Preparing for practice*^2^ (PfP) guidance from September 2025 for incoming cohorts. The SPF further refines the regulator's move, from setting mainly technical competencies to be achieved before a student qualifies, towards a more integrated set of required professional outcomes. In common with PfP, the SPF sets out the required learning outcomes for graduating dentists in four domains, but these domains have been updated from ‘clinical', ‘communication', ‘professionalism' and ‘management and leadership' to ‘clinical knowledge and skills', ‘interpersonal skills', ‘professionalism' and ‘self-management'. A further change incorporated into the SPF, compared to PfP, has been the physical separation of behaviours from the learning outcomes. The reason for this separation is recognition by the GDC that some of the behavioural learning outcomes required in PfP could not be objectively assessed. Therefore, the SPF tries to align the requisite behaviours to the learning outcomes they underpin and now expects providers to objectively assess the learning outcomes, but only provide evidence that the behaviours are being demonstrated throughout the programme. The behaviours included in the SPF should be welcomed because they include crucial psychosocial skills, including insight, adaptability, wellbeing, personal growth, and accountability, which are essential for learning, professional development and personal wellbeing. However, the risk is that if a superficial ‘bolt on' approach is taken to their development by education providers, the consequences to the profession could be significant because these essential psychosocial skills are often lacking in students. In this manuscript, we review the evidence over why these psychosocial skills are essential, why they are often underdeveloped in students, and propose a framework to support their meaningful development within dental curricula.

## Behaviours are essential for learning

**Fig. 1:**
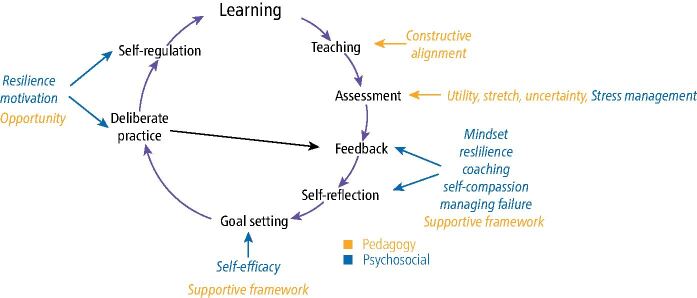
Illustration of the key elements of the learning cycle indicating the requisite pedagogical and psychosocial factors required for their successful implementation^11^

The ideal outcome of all healthcare (including dental) programmes is to produce graduates who can serve the needs of their patients for the benefit of both the individual and wider society through being competent, caring and professional. However, achieving this ideal outcome is very challenging because effective learning requires a synergy between cognitive and psychosocial elements. Within a curriculum, these elements that support learning can be represented as a learning cycle (Fig. 1).

Within this cycle, learning begins through using appropriate and effective teaching that needs to be constructively aligned with learning outcomes and the proposed tools of assessment.^3^ The assessment design should have appropriate utility (ie be valid, reliable, engender the right educational impact, be feasible and acceptable to stakeholders).^4^ In addition, assessment should stretch the students,^5^ be sufficiently sophisticated to embrace uncertainty, contribute to ‘assessment for learning' to support reflection,^6^ and be used in the ‘assessment of learning' to support progression decisions. The results of good assessment should also provide the focus for feedback, self-reflection and goal-setting. However, meeting these goals requires deliberate and organised practice across multiple contexts and difficulties to ensure appropriate development.^5^ With sufficient effort, time and focus, deliberate practice will lead to the development of schema in the long-term memory,^7^ allowing automation. Evidence of automation would signify changes in self-regulation have taken place^8^ and confirm that learning has become embedded in the individual.

However, it is much easier to state learning outcomes and required behaviours than it is to design curricula, assessments and methods of monitoring to meet them. Therefore, it is not surprising that data suggest that many health education programmes are likely to fall short of achieving the ideal outcome, for reasons that may include:

- Assessment approaches that are not sufficiently sophisticated to predict real-world practice through the separation of knowledge and skills,^9^ combined with reductionist approaches for assessing clinical skills, to suggest objectivity^10^
- A teaching and assessment design that actively fosters poor psychosocial development, which can present as task avoidance, a focus on grades, cheating and challenge to feedback.^11^^,^^12^

One solution to improve the chances of producing competent, caring professional graduates who can serve the needs of their patients is to design curricula that actively develop, foster and embed psychosocial skills.

## Understanding our current generation of students

For dental students and trainees, to engage fully with the learning cycle (Fig. 1), and as a result develop both clinically and academically, there is a prerequisite to have also developed a range of psychosocial skills such as motivation, self-regulation, self-efficacy, resilience and management of failure.^13^^,^^14^^,^^15^^,^^16^ The foundations of these skills are usually developed during childhood and adolescence^17^ and therefore variations in the nature or magnitude of the developmental experiences encountered in childhood may consequently affect the acquisition of these psychosocial skills.

Many current undergraduates were born between 1995-2010 and therefore belong to Generation Z.^18^ Due to a range of cultural factors, these individuals have grown up in a very different environment to those of previous generations^12^ and these differences may go some way to explain some of the consequent challenges in medical education that have been highlighted recently.^19^ Data suggest that societal and economic factors can have a demonstrable effect on a person's psychosocial development^20^ and these factors could impact upon their ability to flourish as a student, particularly in curricula that were designed by previous generations.

As a consequence of ubiquitous social media, accessed from an extremely early age, that has encouraged unhealthy perfectionism, and the social and psychological consequences of COVID-19, the current generation of dental students (Generation Z) has not had the same opportunities as their predecessors to fully develop autonomous skills such as risk assessment, communication, negotiation, and dealing with conflict,^21^ which are all essential skills required of a healthcare professional. Therefore, educators need to actively consider the social, educational and economic factors that have affected the development of this generation into early adulthood, as well as their potential consequences. Crucially, within healthcare programmes, although there is currently much emphasis on communication and professionalism, there are currently no published curricula outlining the psychosocial skill development required to support the training of healthcare students.

## Theoretical and practical considerations for a psychosocial curriculum

Psychosocial skills, like clinical skills, require development and their development relies on interaction between the person (a student in this case), the environment and their behaviour in the learning setting.^22^ These skills interact to foster the cognitive, emotional and behavioural repertoire of self-management skills during learning and beyond.^23^ Therefore, developing self-regulated learning requires robust planning, action and reflection.^24^ Consequently, successful planning will also need to form a key aspect of curriculum design, with a team of educators focused on supporting learners towards autonomy.^25^

A comprehensive healthcare curriculum that is fit for purpose should not only develop academic and technical skills but also include staged support that fosters psychosocial development by exposing students to experiences that underpin the learning of these skills (Fig. 1). Ideal teaching would support the student in developing a combination of thought, emotions and a repertoire of behavioural skills which demonstrate that they can meet thresholds. These would allow the educators to judge that the student can transition subsequently to practise as a fully rounded, early-stage professional. This teaching is likely to require explicit instruction in principles of self-management during students' early experiences, combined with authentic opportunities for their practice and a transition where learners take increasingly more ownership of their self-management during the later stages of their education.

The development of student autonomy requires that educators realise that current students are likely not to be inherently self-regulators, and therefore they should be supported to negotiate three broad phases of self-management:^24^

- Planning - in setting effective goals that support students to achieve in ways that play to their strengths and align them with their values and those of the profession. Goals set in this phase should be structured to create the conditions that motivate students towards action^26^
- Action - supporting self-control, motivation and persistence when striving towards goals.^27^ Developing these behaviours through coaching conversations and low-stakes opportunities for development will promote discussions that encourage student self-monitoring and course correction during task performance, as well as support the development of automaticity in task reflection^25^
- Reflection - educators should provide balanced feedback that provides a sense of advice which encourages students to identify what is working, as well as make larger corrections during the next phase of their learning when setting future goals.^28^^,^^29^ Initially, the role of educators is to encourage self-reflection and to be responsive to learner needs, while building appropriate relationships in supporting behavioural change.

Implicit in each of these phases is the development of student self-efficacy.^22^ Self-efficacy speaks to the learner's confidence and expectation that they can achieve specific goals.^25^ Further, developing student autonomy will encourage greater independence in managing and negotiating each phase of self-regulation.^27^^,^^30^ The success of a psychosocial curriculum could be measured by the extent to which the students take greater strides towards competence and confidence in these three phases. If this confidence and a greater sense of expectation is achieved, then students will demonstrate authentic independence in setting goals, acting and reflecting.^24^ This self-moderated confidence will also help the individual expand their skills to recognise where they meet the ‘confidence' element of the GDC standard 7.2.1: ‘you must only carry out a task or a type of treatment if you are appropriately trained, competent, confident and indemnified'.^31^

Developing self-regulation and self-efficacy is not a linear journey. Encountering novel challenges may cause conflicts in learners at all stages, which could undermine their confidence. However, developing the key skills and strategies under discussion will provide a ‘toolkit' of inner psychological resources that learners can draw on and apply to these novel situations. Educators in supportive environments should be mindful of potential trigger points and promote timely conversations that support their students in accessing the tools to manage their journey.^24^

## A proposed syllabus for inclusion within a psychosocial sub-curriculum

In light of the literature discussed, we would suggest that a syllabus that would facilitate the learning of all students from the current generation, and fulfil the SPF, should explicitly address specific elements (Table 1). These psychosocial elements, delivered and assessed by appropriately trained teachers, and reinforced during real-world working, will help to build resilience in future dental professionals, which will allow them to manage the care needs of their patients while maximising their own psychological wellbeing.

**Table 1:** Proposed syllabus for a psychosocial sub-curriculum for dental students

**1. Values and strengths** Supporting motivation Establishing purpose and meaning Building relationships Enhancing wellbeing Belonging Decision-making	**2. Motivation** Self-efficacy Autonomy Belonging Mindset Hope Deliberate practice
**3. Goals** Setting specific and challenging goals (c.f. SMART) Performance versus learning goals Stretch goals Personal/academic/clinical goals	**4. Feedback and reflection** Acceptance of feedback Utility of feedback Delivery of feedback Models of reflection Reflection versus rumination Support framework
**5. Managing self** a) Wellbeing Presence/mindfulness Self-compassion Flow b) Building confidence c) Resilience Coping with stress/uncertainty Fear of failure Managing shame d) Emotional regulation	**6. Working with others** Building positive relationships Psychological safety Equality, diversity and inclusion Emotional intelligence Management and leadership Challenging conversations

However, several practical difficulties can be foreseen:

1. There is no pre-university assessment of these aspects of development; therefore, each student will enter dental education with their own individual set of skills. In dental admissions processes, academic achievement is often the major tool in initial selection, followed by other assessments (eg University Clinical Aptitude Test) and selection interviews that tend to use a panel or multiple mini interview systems.^32^ Consequently, many educators will try to select a candidate who they consider will best fit into their institution's programmes, without explicitly assessing psychosocial skills. The only currently mandated part of the process is ‘values-based recruitment', where it must be shown that each candidate is selected against the values stated in the NHS Constitution.^33^ While strengths-based selection has started to be introduced within healthcare selection processes^34^ (specifically nursing and midwifery), this has not yet been adopted within dental admissions processes.^35^ This limitation in selection processes means that, without very careful curriculum design and student education, many students will likely have great difficulty in adjusting to these new areas being monitored, and possibly, in their eyes, will have ‘failed'
2. The relevant curriculum design skills and teacher expertise within the school may be lacking, so there may be a significant need for staff training
3. Educators will need to work out how to monitor and assess their students' development in these areas (Fig. 1), given that this will often be non-linear and dependent upon the individual's life experience. A starting point for dental schools may be to:
  - Initially, group the SPF behavioural outcomes into a set of themes (Table 2) that also align to psychosocial areas (identified in Table 1)

**Table 2:** Proposed domains to aid monitoring of *The* *safe practitioner* framework behavioural outcomes (identified in italics) linked to psychosocial skills

**Lifelong learning**	**Responsibility**	**Inclusivity**	**Integrity**	**Collegiality**
Goals, feedback and reflection, motivation	Values and strengths, managing self, working with others			
Adopt an evidence-based approach to clinical practice *C(B)1*	Demonstrate personal accountability to patients, etc. *P(B)14*	Demonstrate cultural competence, accepting and respecting the diversity of patients and colleagues *P(B)3*	Act within the legal frameworks which inform personal behaviour, the delivery of healthcare etc *P(B)10*	Work…with colleagues to develop…an effective and supportive environment which promotes the safety…of the patient and dental team *P(B)15*
Accurately assess your own capabilities and limitations in the interest of high-quality patient care and seek advice from supervisors or colleagues…*S(B)1*	Speak up to protect others from harm *P(B)5*	Treat your patients, members of the public and your colleagues with dignity, respect and without discrimination *P(B)1*	Manage and refer/delegate work according to the scope of practice of…the dental team, in line with competence and professional practice *I(B)6*	Respect the roles of dental and other healthcare professionals in the context of learning and working… *I(B)2*
Develop and maintain professional knowledge and competence. *S(B)5*	Demonstrate own professional responsibility in the development of self *S(B)4*	Communicate with care, compassion, empathy and respect in all professional interactions… *I(B)1*	Act with integrity and ensure your actions maintain the trust of colleagues, patients and the public… *P(B)12*	Contribute positively to the healthcare communities of which you are a part *P(B)17*
Demonstrate appropriate continuous improvement activities. *S(B)6*	Lead, manage and take professional responsibility for the actions of colleagues and other members of the team… *P(B)16*	Recognise personal assumptions, biases and prejudices and manage the impact of these … *S(B)2*	Recognise…contextual factors on the healthcare environment and patient safety and manage this… *S(B)3*	Demonstrate effective team-working *I(B)3*
Effectively manage your own time and resources. *S(B)9*	Support patients to make informed decisions about their care, making their interests your first concern *P(B)2*	Proactively address discriminatory language, behaviour and microaggressions … *P(B)13*	Act in accordance with current best practice guidelines *P(B)8*	Contribute to your team in providing dental care for patients *I(B)4*
Demonstrate engagement with systems and personal strategies…promote and maintain physical and mental well-being *S(B)7*	Recognise…how to take action if wellbeing is compromised to a point of affecting and individual's role … *S(B)9*	-	Act in accordance with national and local clinical governance and health and safety requirements *P(B)9*	Take a patient-centred approach to working with the dental and wider healthcare team *I(B)5*
-	Provide the best possible outcome for your patients by using your knowledge and skills, …*P(B)4*	-	Maintain contemporaneous, complete and accurate patient records … *P(B)11*	-
-	Raise concerns where appropriate about your own or others' health, behaviour or…performance *P(B)6*	-	-	-
-	Comply with systems and processes to support…patient care *P(B)7*	-	-	-
  - Monitor students' progress in each of these themes throughout their programme
  - Ensure that there are real-world consequences for not demonstrating readiness in these themes.

The obvious challenge is how to monitor development in the themes because it will require authentically operationalising the teaching to embed the skills (Table 1), combined with sophisticated approaches to triangulate and integrate the data^11^ over how well the skills are being used to act as the driver for timely feedback, self-reflection and the setting and demonstrable meeting of goals (Fig. 1).

An attractive solution to the problem is to embrace programmatic assessment,^11^^,^^36^ which develops skills using low-stakes ‘learning moments' to support good educational impact^5^ and assesses the current level of development through the integration and triangulation of data from multiple sources and contexts to make higher stakes decisions. Therefore, programmatic assessment fully aligns to the learning cycle (Fig. 1) and can establish developmental progression when deployed within a carefully created learning design.

To illustrate a programmatic approach, consider the lifelong learning theme (Table 2) - evidence for each of the associated SPF behaviours could be gathered from a series of longitudinal low-stakes activities, each of which have been subject to timely and appropriate feedback, as well as opportunities for student development. This evidence could include: appropriate written assessment questions from multiple different assessments; work-based assessment; response to feedback; setting and meeting goals; timeliness of completion; and fruitful engagement with teaching tools for personal development. When combined, this would provide a rich source of contextual data, which could be used to support further reflection and development, as well as inform an evidence-based readiness decision.

However, it should be noted that programmatic assessment does not come without its challenges, which include aspects of implementation,^37^ difficulties with staff and students not understanding the difference between low- and high-stakes assessment,^38^^,^^39^ and complexities around data triangulation, integration and decision-making with such large data sets.^39^ Therefore, any adoption of programmatic approaches requires careful thought, compatible curriculum design and buy-in from all stakeholders.

## Conclusion

Available evidence suggests that educational institutions need to embrace psychosocial development within their evidence-based healthcare programs. An integrated psychosocial curriculum would not only maximise the potential of the future dental workforce but also serve as a constructive response to the SPF. Overall, the identification and expression of psychosocial elements within the learning design should support and enhance the existing curriculum by promoting the continued growth, development and mental wellbeing of students (and possibly staff), which should lead to improved student outcomes and patient care.

## Data Availability

Not applicable.
